# A Self-Learning Mechanism-Based Approach to Helicopter Entry and Departure Recognition

**DOI:** 10.3390/s22207852

**Published:** 2022-10-16

**Authors:** Zonglei Lyu, Xuepeng Chang, Wei An, Tong Yu

**Affiliations:** 1 College of Computer Science and Technology, Civil Aviation University of China, Tianjin 300300, China; 2 Key Laboratory of Smart Airport Theory and System, Civil Aviation University of China, Tianjin 300300, China

**Keywords:** self-learning mechanism, automatic data selection, pseudo clustering, image classification

## Abstract

In order to accurately record the entry and departure times of helicopters and reduce the incidence of general aviation accidents, this paper proposes a helicopter entry and departure recognition method based on a self-learning mechanism, which is supplemented by a lightweight object detection module and an image classification module. The original image data obtained from the lightweight object detection module are used to construct an Automatic Selector of Data (Auto-SD) and an Adjustment Evaluator of Data Bias (Ad-EDB), whereby Auto-SD automatically generates a pseudo-clustering of the original image data. Ad-EDB then performs the adjustment evaluation and selects the best matching module for image classification. The self-learning mechanism constructed in this paper is applied to the helicopter entry and departure recognition scenario, and the ResNet18 residual network is selected for state classification. As regards the self-built helicopter entry and departure data set, the accuracy reaches 97.83%, which is 6.51% better than the bounding box detection method. To a certain extent, the strong reliance on manual annotation for helicopter entry and departure status classification scenarios is lifted, and the data auto-selector is continuously optimized using the preorder classification results to establish a circular learning loop in the algorithm.

## 1. Introduction

The automatic acquisition of aircraft entry and departure information from airport video surveillance by object detection methods represents an important application of artificial intelligence technology in airport operations, and an important part of intelligent civil aviation construction. In the 14th Five-Year Plan for Civil Aviation Development released in 2021, it is clearly stated that China needs to vigorously develop general aviation. Compared to transport airports, general aviation airports have a smaller footprint, simpler structures, and more indirect operating rules. The operational rules used to aid object detection at transport airports are not fully applicable at general aviation airports, especially for the helicopter entry and departure process, which differs markedly from that for fixed-wing aircraft [1,2]. Therefore, the recognition method for helicopter entry and departure positions at general aviation airports has strong research and application value [3,4].

Taking the entry and departure scenario of fixed-wing aircraft as an example, the process of recognizing the entry of fixed-wing aircraft at transport airports is based on the detection of the aircraft and its position information by the object detection model, followed by methods such as optical flow to recognize the movement of the aircraft, which is considered to be in the entry state if the aircraft is in position and is stationary [5]. However, recognition based on position and movement state for entry is not applicable to helicopters. The helicopter will hover when it is in the entering position, and even when it is at zero altitude with the landing gear not fully supported, at which point, the helicopter is also relatively stationary, it will not yet have completed its entry. In addition, the propeller will remain rotating for a period of time after the helicopter has entered the position, so the determination of the movement status using the optical flow method with the propeller alone will be somewhat biased [6].

Despite significant advances in research into artificial intelligence systems, knowledge learning in AI still relies partly or entirely on human supervision. In order to enhance the learning capability of AI and enable AI to establish a fundamental connection between knowledge input and output (or action/experience), self-learning and sustainable learning are gradually becoming a source and motivation for the development of autonomous AI methods, and moreover, represent a fundamental theoretical problem to be addressed by the next generation of AI algorithms and models [7]. To achieve autonomous AI that adapts to the environment, data self-generation and data self-selection are among the goals that need to be addressed in AI automation [8,9].

Numerous approaches that have been proposed for helicopter flight status recognition have led to results with minor errors, especially when using computer vision-based techniques. Xiong et al. [10] proposed a flight status recognition method based on SVM in order to solve the problem of low recognition rate due to insufficient training samples. In [11], Wang et al. proposed a flight condition recognition method based on random forest. In [12], Gao proposed a flight condition recognition method based on BP Neural Networks for the problem of low recognition rate. In [13], Li et al. proposed a flight condition recognition method based on the pre-classification mechanism and GRNN. Although all of the above algorithms achieved some improvements in recognition accuracy, they were hindered by the limitations of the underlying algorithm, and did not achieve the accuracy required by the airport. In [14], Li et al. used a deep learning-based approach to aircraft landing gear status detection to address the problems of safe aircraft landings. However, the above methods do not address the problem of low accuracy due to insufficient training samples. To address the problem of low recognition accuracy, Feng [15] proposed a flight status recognition method based on an airborne vibration monitoring system. Nikola et al. in [16] and Wang et al. in [17] proposed the use of laser scanning based on multi-sensor technology for recognition. However, due to the cumbersome process of multi-sensor arrangement and expensive equipment, this approach is not suitable for general aviation airports. In this regard, we want to introduce a self-learning mechanism to solve the problem of insufficient data, and we use the selected excellent image classification module for the recognition of the position state of the helicopter.

Considering the importance of self-learning in solving practical problems, this paper takes the helicopter position information extracted from object detection as the starting point of the self-learning mechanism, realizes the automatic generation and evaluation of helicopter entry and departure data, selects the appropriate image classification module for classification from the evaluation results of the generated data, then optimizes the data generation in the later order using the results of the previous order classifications, and updates this in a cycle to realize a self-learning mechanism for helicopter entry and departure status recognition.

The main contributions of this paper are as follows:

1. The lightweight YOLOv5s algorithm is applied for the fast detection of helicopter video, using the detection bounding box to calculate the relative position of the helicopter, thus generating a collection of raw image data as input to the self-learning mechanism;
2. The self-learning mechanism constructed in this paper can select and update the image classification module, which establishes a dynamic cycle of overall self-learning of the algorithm, and promotes the cyclic optimization of model learning. Taking the helicopter entry and departure state as an example, the self-learning mechanism built in this paper has a certain capacity for generalization and can solve the entry and departure recognition problem of most relatively rigid bodies in a fixed area.
3. Building an Automatic Selector of Data (Auto-SD) and Adjustment Evaluator of Data Bias (Ad-EDB) to automatically label the helicopter motion state with the original data set generated from a priori knowledge and object detection. The algorithm combines a priori knowledge and the original data set generated from object detection to automatically annotate the helicopter motion status, adjust and evaluate the accuracy of the labels, select the best matching image classification module for training, and finally achieve the status recognition of helicopter entry and exit positions.

**Figure 1:**
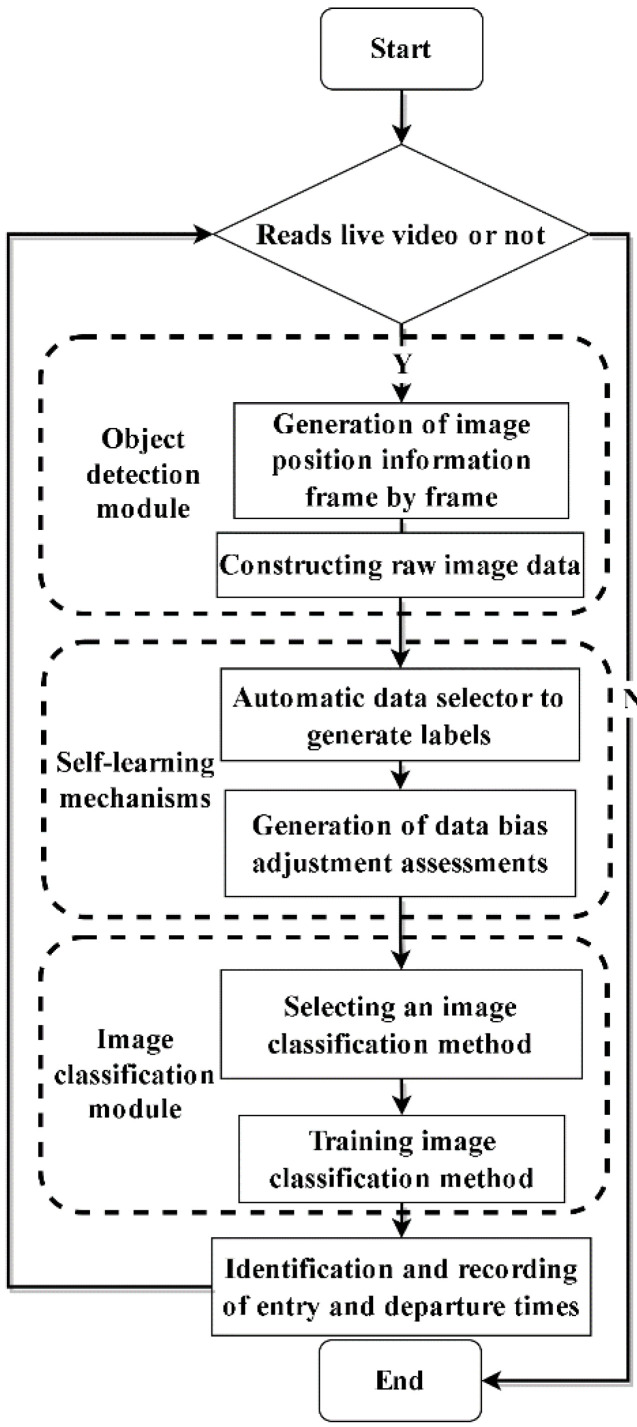
Overall workflow diagram for this paper.

The overall workflow is shown in Figure 1.

## 2. Related Work

### 2.1. Object Detection

The self-learning mechanism requires the use of object detection methods to quickly acquire the bounding boxes of detected helicopters in the working environment of a helicopter ramp in order to complete cropping as raw data for subsequent data self-generation and data self-selection. With the increase in GPU computing power and the research of neural networks in recent years, object detection has become a hot spot in global artificial intelligence research. Most of the current mainstream object detection methods are based on convolutional neural networks, and in recent years, two main categories have been formed: candidate region-based and regression-based [18]. Candidate region-based object detection methods, also known as two-stage methods, divide the object detection problem into two stages; one is to generate candidate regions, and the other is to put the candidate regions into the classifier to classify and correct the position. The most common two-stage methods are RCNN [19], Fast-RCNN [20], and Faster-RCNN [21] series; however, the two-stage methods have the problems of slow detection speed and complex parameters, and are not applicable to the object detection method used in this paper. Regression-based object detection methods, also known as one-stage methods, perform regression directly on the predicted target object. Among the most common one-stage methods are SSD [22] and the YOLO series [23,24,25]. One-stage methods are significantly faster in terms of detection compared to two-stage methods, but the number of parameters is still high.

In order to obtain the bounding box of the detected helicopter quickly, the one-stage method YOLOv5s is chosen as the basis for this paper, and the number of parameters is reduced by modifying the network backbone structure to improve the detection speed.

### 2.2. Image Classification

Image classification is one of the fundamental tasks in computer vision, and thanks to the developments made in deep learning, deep convolutional neural networks have become prominent in image classification tasks [26]. Compared to traditional image classification algorithms that extract features manually, convolutional neural networks use convolutional operations to extract features from input images, effectively learning feature representations from a large number of samples with greater model generalization capability [27]. In this paper, the existing Residual Networks [28] are selected as the basis for completing image classification in the helicopter entry out-of-place recognition task.

The helicopter entry and departure recognition problem is transformed into a post-detection image classification problem after processing by the object detection module and the self-learning mechanism, and the classification model is expected to focus on the surrounding background features, the landing gear, and the ground contact features. Considering the specificity and real-time nature of the aviation safety field, which requires a certain number of model parameters and a degree of computational speed, and the fact that the ResNet18 model can tolerate the noise of the label data generated by the self-learning mechanism to a certain extent, the ResNet18 model is applied to classify the images. The main difference between ResNet and other networks is the introduction of a residual function in the convolutional neural network. This has the advantage of alleviating the gradient disappearance problem associated with increasing depth in a convolutional neural network and makes the ResNet network easy to optimize, i.e., the accuracy can be improved by increasing the depth of the network. The residual learning module is effective in avoiding the gradient explosion and disappearance problem faced by neural networks when reaching a certain number of layers, further optimizing the performance of deeper networks.

**Figure 2:**
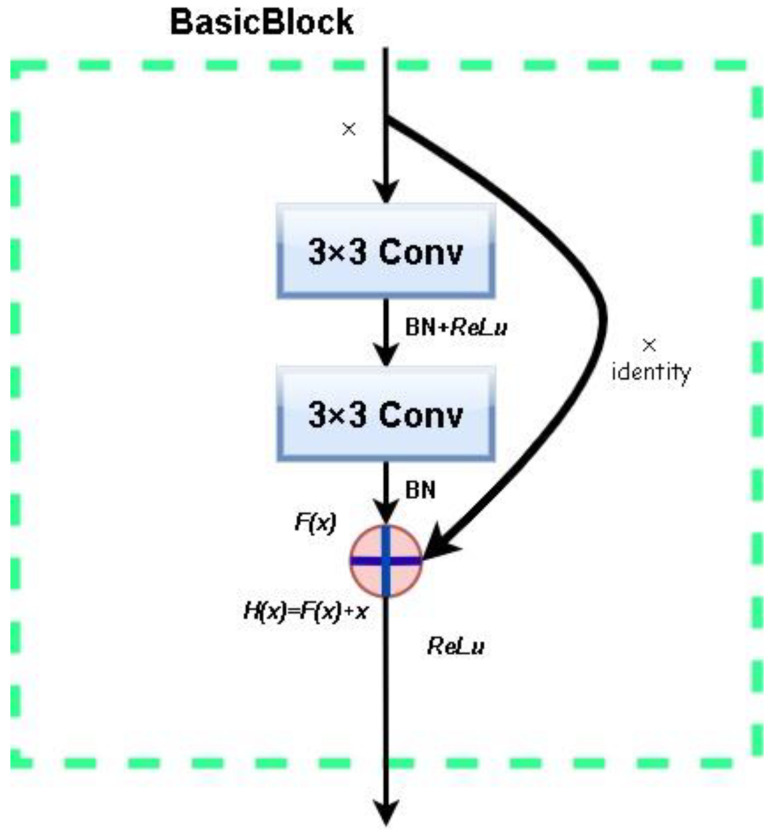
ResNet residual learning module.

The typical structure is shown in Figure 2. x is the input and H(x) is the desired output, which represents the residual function F(x) obtained after a series of processing. The most important feature of the residual network is the introduction of a bypass connection with a constant mapping relationship, so that the actual output H(x) of the residual learning module is the sum of F(x) and the input x of the residual block, and the residual network is converted from learning the mapping from x to H(x) to learning the mapping from F(x) to 0. This reduces the training parameters and computational effort, making the model training faster and more effective.

### 2.3. Helicopter Landing and Take-Off Operating Rules

**Figure 3:**
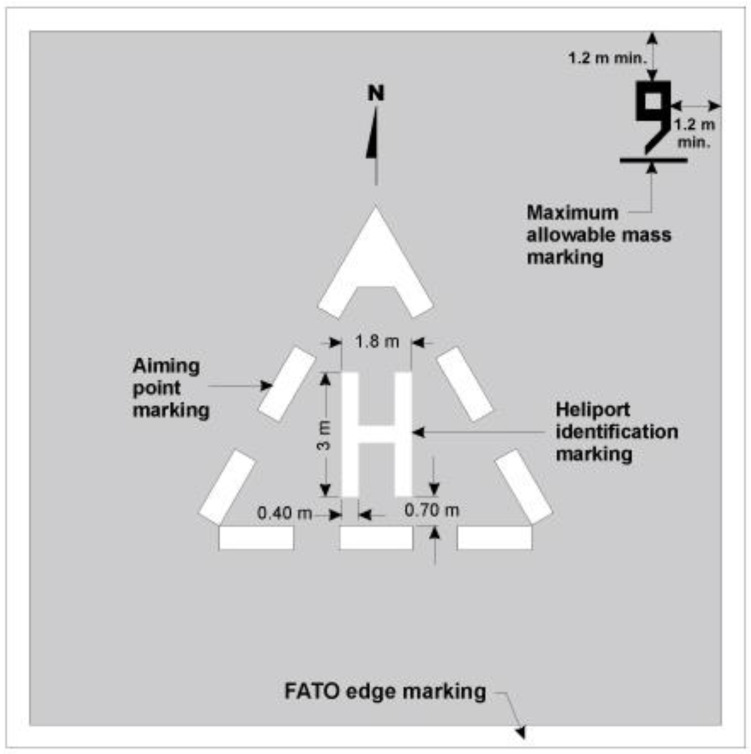
Heliport identification markings map.

Relatively rigid bodies are objects that change shape to a small degree when in motion and subjected to forces, and can be neglected when studying the entry and departure status of an object. For example: cars, helicopters, yachts, etc. In this paper, a priori knowledge is implanted to build Auto-SD based on the specificity of relatively rigid bodies, general aviation, and helicopter parking [29]. The vertical take-off and landing of the helicopter allow for the detection of the bounding box without significant drift or jitter during the transition of the entry and departure status. As shown in Figure 3, the FATO is the final approach and take-off area, with the helicopter’s entry and departure position operating in the FATO. With the exception of hospital heliports, heliport identification signs should be set in the center of the aiming point markings, i.e., the helicopter’s entry and departure process is indicated and guided by the heliport identification sign “H” on the helipad [30]. The “H”, which serves as the identification mark of the heliport, allows the pilot to better observe the helicopter in the air and to correct the landing point, and the helicopter parks in the area clearly marked with an H.

## 3. Self-Learning Mechanism-Based Entry and Departure Recognition Model

### 3.1. The Lightweight YOLOv5s Algorithm (YOLOv5s-RMV3S)

**Figure 4:**
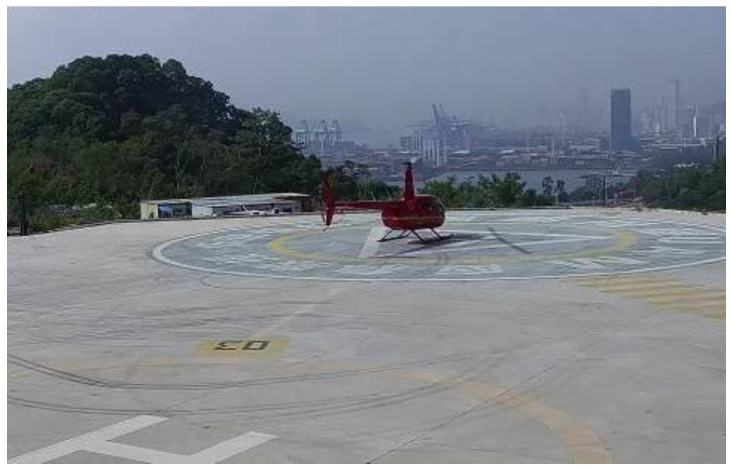
Helicopter to be tested.

As shown in Figure 4, the helicopter to be detected is relatively rigid and has a large target nature, so the detection accuracy is less affected by the number of model parameters and the complexity of the model during the detection process. However, due to the special characteristics of airspace management and air traffic control, the model needs to have a fast computing speed and low computing power requirements. In this paper, the backbone network of YOLOv5s is replaced by the backbone network of Mobilenetv3-small, a lightweight neural network with few parameters, high speed, low memory consumption, and feature extraction performed by depth-separable convolution instead of the original convolutional layer.

**Figure 5:**
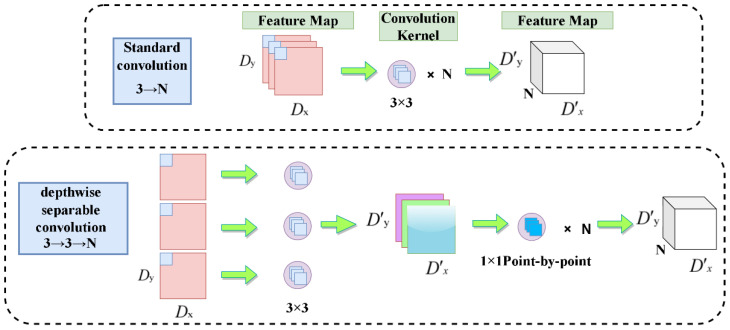
Comparison of standard convolution and depth-separable convolution.

The fundamental algorithm of Mobilenetv3 [31] substitutes a depthwise separable convolution for conventional convolution, as seen in Figure 5.

A $D_{x}\times D_{y}\times 3$ feature map is used as the input, and a ${D^{\prime }}_{x}\times {D^{\prime }}_{y}\times N$ feature map is used as the output following convolution using a 3×3 convolution kernel. The depthwise separable convolution first convolves three 3×3 convolution kernels with each channel of the input feature map to obtain a feature map with the input N 3×3 channel equal to the output channel, and then convolves this feature map with N 1×1 convolution kernels to obtain a new feature map with N channels. This method is similar to the standard convolution process. With depthwise separable convolution, the number of parameters needed to obtain comparable results to regular convolution may be considerably decreased.

Replacing the backbone network of YOLOv5s with the backbone network of Mobilenetv3-small reduces the number of parameters while increasing the computing speed and significantly reducing the need for computing power. However, from the practical perspective of helicopter detection, the Mobilenetv3-small can still be adapted to the needs of this paper due to the specificity of the detection object and the application scenario. Firstly, in the Conv3BN layer, the convolution step size is changed from 2 to 1. In this layer, the feature scale is maintained and the number of channels is extended to accommodate the original helicopter features and region size. Due to the modification of the Conv3BN layer, the third and fourth layers have large redundancy, and the extracted features are similar. The modified Mobilenetv3 backbone network is referred to as the RMV3S module in this paper.

### 3.2. Training Algorithm for Entry and Departure Recognition Models

The training algorithm for the entry departure recognition model is shown in Algorithm 1. Firstly, each frame of the cropped image within the video and its corresponding bounding box information for each frame are obtained through the object detection module. The frame number of the detected video image is recorded, and the difference between the frame number and the interval time of the video is compared by traversal; each frame is collated to match the corresponding entry and departure training video segment. Auto-SD and Ad-EDB methods are applied to generate and evaluate the image labels, and a suitable image classification module is selected according to the evaluation results and rules. The selected image classification module is used for training and building the in-position recognition model.
**Algorithm 1** Entry and Departure Model Training Algorithm**Input: video V = <f1,f2,…,fn>, image classification algorithm set SUM_M, video interval time *η*, minimum tolerable noise rate *ε*.****Output: Entry and departure recognition model M_t_.**1: $R_{i}=\{i\}\times OD(f_{i}),\forall i=1,\ldots ,n$, where $OD(f_{i})$ indicates a lightweight object detection module2: Define S to store video image frame numbers and information after video image detection, $S=\overset{n}{\underset{i=1}{\cup }}R_{i}$3: The video image frame number set A=S.colomn(1), where colomn(1) represents the extraction of the first component of the set4: $\forall j\in A,\alpha_{j}=1\left(\exists k\in A(j-k<\eta \right.\left.)\right)$,5: $\varphi_{j}=\sum_{k=1}^{j-1}\alpha_{j}$6: ∀1≤m≤n, paragraph m entry and departure training video $S_{m}=\{s_{j}|\varphi_{j}=m\}$7: All generated tag collections SUM_TAG =$\underset{1\leq m\leq n}{\cup }\mathrm{Auto}-\mathrm{SD}(s_{m})$, where Auto-SD indicates the generated tag method; see Section 3.2 for details8: All generated tag noise rates *δ* = Ad-EDB(SUM_TAG), where Ad-EDB denotes the tag conditioning evaluation method; see Section 3.3 for details9: Image classification algorithm M = $\underset{size}{arc\min}\{M_{i}\in \mathrm{SUM}\_M|\delta <\varepsilon \}$10:  Entry and departure recognition model M_t_ = Train(M,S,SUM_TAG)

The algorithm in this paper obtains the required entry and departure recognition model by automatically processing the video and constructing the method in conjunction with the video processing results. The first two of these steps correlate the detection results of the video image with the corresponding frame number, and then store the correlation results. Steps 3–6 use the correlation results to select the start frame of the in–out training video for each segment of the video, and slice the video into multiple in–out training video segments. Step 7 uses Auto-SD to select the core still array for each in–out-of-position training video, and automatically labels the images with the corresponding motion or still labels according to the stored results of the core still array, as described in Section 3.3. Step 8 uses Ad-EDB to optimize all tags, and after optimization, a quality assessment of all tags is carried out, as detailed in Section 3.4. Step 9 selects the image classification algorithm that meets the evaluation results and has the smallest algorithm model in the image classification algorithm set based on the evaluation results. The final step uses the generated labels and images to train on an image classification algorithm to obtain the final entry and departure recognition model.

### 3.3. Automatic Selector of Data (Auto-SD)

In order to accurately identify the entry and departure status of helicopters, it is necessary to accurately label and judge the stationary or motion status of helicopters in the video stream. This paper proposes an Auto-SD based on a pseudo-clustering method for filtering and generating information on the stationary or motion status of helicopters.

For the identification of entry and departure states, the stationary state of the helicopter is not absolute, and in situ and contextual information becomes the key to determining the stationary or moving state. For example, when the helicopter is fully on the ground and the propeller is rotating, the entry and departure state recognition problem identifies this as a stationary state. This paper introduces the object detection module mentioned in the above work to obtain the position information of the helicopter from the video stream, obtain the relative bounding box of the helicopter, and thus generate an unlabeled original image of the helicopter with obvious in-place information and precise contextual information. Due to the temporal continuity of the video, the subsequent processing and classification of the bounding box can theoretically lead to an approximate distribution interval of stationary or moving states. However, the bounding box often jitters during detection, resulting in inaccurate relative position results, and can only be used as reference information for state classification, while the image classifier trained with the reference information is the accurate method for identifying helicopter entry and departure states. This paper therefore introduces Auto-SD to provide accurate and automatic data support for the subsequent image classifier.

**Figure 6:**
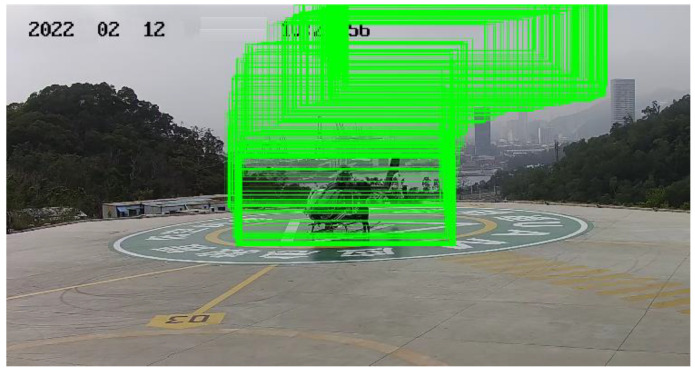
Bounding box effect with stillness as background.

As shown in Figure 6, it can be concluded with respect to the operational rules for helicopter take-off and landing that the rate of overlap of the bounding boxes of the helicopter at rest in each frame is very high, i.e., the IOU (Interaction Over Union) is very high. In contrast, the IOU values in motion and at rest are relatively low, or may not even overlap.

Due to the temporal continuity of the video image, the detected information on the position of the bounding box can subsequently provide the basis for the construction of Auto-SD. We assume the existence of the original video data V; after the object detection module $f_{ob}(x)$ processing, $f_{ob}(V)$, we obtain the frame-by-frame information of the bounding box position $P_{i}=[x_{i},y_{i},w_{i},h_{i}]$; $x_{i},y_{i},w_{i},h_{i}$ represent the horizontal coordinates of the lower left corner of the bounding box, the vertical coordinates of the lower left corner of the bounding box, the width of the bounding box, and the height of the bounding box, respectively, where i∈[1,n] and i is the integer, and n represents the total number of frames of the original video data V, as shown in Equation (1).

(1) $$f_{ob}(V)=P_{i},i\in [1,n]\&i\in Z$$

As shown in Equation (2), the boundary frame position information $P_{i}$ obtained by detection is subjected to IOU calculation two by two to obtain $a_{j}^{i}$, and each item of boundary box position information $A^{i}$ is obtained with its corresponding value array $A^{i}$, where array $A^{i}$ contains n calculated IOU values $a_{t}^{i}$, t∈[1,n] and the values $a_{t}^{i}$ range in [0,1].

(2) $$\begin{array}{l} a_{j}^{i}=IOU(P_{i},P_{j}),j\in [1,n]\&j\in Z\\ A^{i}=[a_{1}^{i},a_{2}^{i},\ldots ,a_{t}^{i},\ldots ,a_{n}^{i}],a_{t}^{i}\in [0,1]\&a_{t}^{i}\in R \end{array}$$

We set the threshold value β and set $b_{t}^{i}$ to represent the state information. If $a_{t}^{i}$ is greater than β, we set the state $b_{t}^{i}$ to 1, while if it is less than or equal to it, we set the state $b_{t}^{i}$ to 0. After the threshold judgment classification, the array $A^{i}$ corresponding to any $P_{i}$ is converted into a relative state $b_{t}^{i}$ array $SA^{i}$ with other location information, as shown in Equations (3) and (4).

(3) $$b_{t}^{i}=\left\{\begin{array}{c} 0,a_{t}^{i}\leq \beta \\ 1,a_{t}^{i}>\beta \end{array}\right.$$

(4) $$SA^{i}=[b_{1}^{i},b_{2}^{i},\ldots ,b_{t}^{i},\ldots ,b_{n}^{i}],b_{t}^{i}\in [0,1]\&b_{t}^{i}\in Z$$

The number of access $b_{t}^{i}$ to 1 in $SA^{i}$ is much greater than the number of access $b_{t}^{i}$ to 0 due to the relatively fixed and stationary states of the selected bounding boxes relative to the rigid body mooring position almost overlapping. As shown in Equations (5) and (6), traversing $SA^{i}$ in the range [1,n] identifies the largest number of access states as 1 for the core stationary position array $SA^{k}$, k unique and k∈[1,n].

(5) $$f(i)=\sum_{t=1}^{n}b_{t}^{i}$$

(6) $$k=\arg\max\left(f(i)\right)$$

For a label with a status of 1 relative to the core rest position label, i.e., when $SA^{k}$ stores $b_{t}^{k}$ of 1, we label image $P_{t}$ as rest label $l_{s}$, while if $SA_{k}$ stores $b_{t}^{k}$ of 0, we label image $P_{t}$ as motion label $l_{m}$.

In the above description of the workflow of Auto-SD, the video passage taken by default in this article is a complete helicopter take-off and landing. However, due to the temporal continuity of video images in real situations, this paper needs to select a suitable time window for the regular slicing of the video. By counting the results of the object detection, the real-time movement of the helicopter in the helicopter position monitoring area can be obtained, and then the length of time for which the helicopter disappears in the position area can be obtained by counting the video frames. Using the length of time for which the helicopter disappears from the area of the aircraft as a measure, the video is logically sliced for subsequent work on Auto-SD.

This paper enables the processing of real-time video to generate helicopter image data with motion or static labels, and the smooth construction of an efficient Auto-SD.

### 3.4. Adjustment Evaluator of Data Bias (Ad-EDB)

In this paper, the object detection module is used to obtain the raw unlabeled helicopter image data, and then Auto-SD is used to obtain the corresponding status labels of the helicopter images, which is an efficient and automatic way to complete the complex image data annotation work. However, due to the small amount of a priori information available during the construction of Auto-SD, the generated label data are biased, and therefore Ad-EDB needs to be constructed to process and evaluate the generated label data.

After the Auto-SD process, the image data adopt a continuous sorted distribution with labels. In the sorted distribution of image data, the image data paragraphs can be recursively nudged through a time-sliding window of three frames. There are eight sorted arrays of label information for the three frames $S=\langle s_{1},s_{2},s_{3}\rangle$. Among these eight types of arrays, arrays with no change in state information or no jump in state information are classified as reasonable arrays in this paper, and there are six reasonable arrays $S_{N}$. Since the state of the helicopter does not undergo sudden jump transitions in the vast majority of cases when $s_{1}⊕s_{2}$ and $s_{2}⊕s_{3}$, this paper classifies arrays with jump changes in state information as anomalous arrays $S_{E}=\langle s_{1},s_{2},s_{3}\rangle$. In this paper, $s_{2}$ in $S_{E}$ is defined as an exception frame. As shown in Equation (7), to improve the relative accuracy of the generated data labels, the label information in $S_{E}$ should be optimized. However, it is not universal to rely on the information data of only three frames for optimization, so $S_{E}$ is used as the starting point to extend the labeling information backward, and the subsequent labeling information is counted. If the subsequent labeling information maintains the state of $s_{1}$ and $s_{3}$ in $S_{E}$, then the label information of $s_{2}$ in $S_{E}$ is optimized. After conditioning and processing the generated labels, a small improvement in relative accuracy was achieved.

(7) $$S_{E}=\langle s_{1},\neg s_{2},s_{3}\rangle$$

During the optimization process, the same situation arises wherein the subsequent state label statistics differ from the $s_{1}$ and $s_{3}$ states in $S_{E}$, and cannot be reconciled in the above way. However, the probability of this occurring is small and the error is negligible after the subsequent image classifier processing, so this paper only needs to select a suitable image classifier according to the value of this error.

The automatically generated label data still deviate from the original label data. The $s_{2}$ in all $S_{E}$ was counted by sliding a three-frame time window from the initial start of the data, and the $s_{2}$ count was compared to the total number of data to obtain a value, which was defined as the pessimistic noise rate $R_{g}$. Clearly, it is a pessimistic statistical approach to consider the $s_{2}$ of all $S_{E}$ as wrongly chosen labels. We define the existence of a video V after Auto-SD to generate an array of labels as $S_{V}=\langle s_{1},s_{2},\ldots ,s_{n}\rangle$, where n denotes the total number of frames of the original video data V. In $S_{V}$, the array of tags containing i segment exceptions is defined as $S_{Vi}=\langle s_{i1},s_{i2},\ldots ,s_{im}\rangle ,n\geq m\geq 3,s_{im}-1⊕s_{im}$. For each segment of the anomalous label array to be manipulated, a three-frame time window was slid and calculated in a pessimistic noise rate manner, with all the $\langle s_{i1},s_{i2},\ldots ,s_{im-1}\rangle$ in $S_{iV}$ being anomalous frames, and for $S_{Vi}$, its $R_{g}$ being m−2/m. We define the length of the segment i anomaly label array as $m_{i}$ and the pessimistic noise rate $R_{g}$ of the $S_{V}$ of the whole video, as shown in Equation (8).

(8) $$R_{g}=\frac{\sum_{p=1}^{i}m_{i}-2i}{n}$$

In practice, abnormal tag arrays adjusted to normal may all be in motion or all stationary, and the calculation of the mis-selection rate changes. If $s_{i1}$ of the abnormal tag array $S_{Vi}$ is in motion, we slide the three-frame time window and count $s_{2}$ only for $S_{E}$ where the first frame is in motion. If $s_{i1}$ in $S_{Vi}$ is stationary, with a sliding three-frame time window, we use only the first frame for the stationary state of $S_{E}$ for $s_{2}$ statistics, and the statistics of $s_{2}$ and the total amount of data of $S_{Vi}$ as a ratio to derive the value. The value will be defined as the optimistic noise rate $R_{o}$, and for the whole video $S_{V}$, $R_{o}$ is calculated as shown in Equation (9).

(9) $$R_{o}=\frac{\sum_{p=1}^{i}m_{i}-i}{2n}$$

From the above, $R_{o}\leq R_{g}$, and as the number of anomalous arrays increases, the difference between $R_{g}$ and $R_{o}$ becomes larger, and the existence of the interval $\left[R_{o},R_{g}\right]$ can be used to measure the efficiency and accuracy of Auto-SD. Based on the interval $\left[R_{o},R_{g}\right]$, the noise rate of the generated image labels can be judged. In turn, an image classifier with a tolerant noise rate for processing labeled image data can be selected, constituting an accurate closed loop of the self-learning mechanism.

## 4. Experimental Results and Analysis

### 4.1. Data Set and Experimental Environment

**Figure 7:**
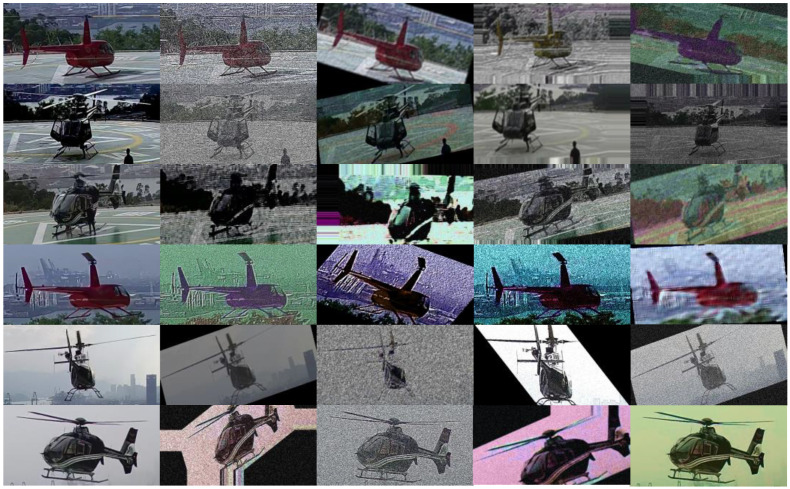
Selected data sets after data enhancement.

This paper collects and selects 31 multi-scene, multi-position, real-time videos of helicopter flight conditions, each 24 h in length, and each containing at least 12 helicopter entry and exit sequences. The object detection module automatically intercepts the recorded video images of the selected helicopter ramp and position as a test set and verification set. In the self-learning module, this paper applies the object detection module and a priori knowledge to self-generate a helicopter state data set based on the video recorded from the selected helicopter ramp and position, and then adjust and process the state labels to ensure the accuracy of the test set, generating a total of 42,989 image data points with state labels. Although the number of image data points is relatively large, the small number of helicopter types owned by the collected heliports results in the high similarity of the detected samples and poor sample quality, which will lead to a lower effect of the trained model and weaker generalization ability. Therefore, this paper uses AugMix data enhancement to process the data and make them more effective and balanced, and part of the data set after data enhancement is shown in Figure 7.

An improved object detection module is applied to detect helicopter video data in order to generate the original image data set. The generated results are validated on multiple live videos of helicopter flight conditions.

**Figure 8:**
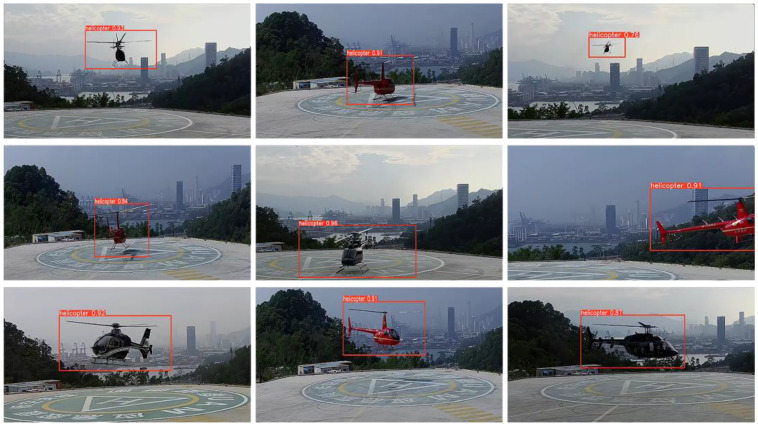
Some of the detection results of the algorithm in this paper.

As listed in Table 1, P stands for accuracy, R for recall, and mAP for mean accuracy. The computational volume of YOLOv5s is 16.4 GB and the model size is 13.7 MB. After improving the backbone extraction network structure using Mobilenetv3-small, and with the channel expansion and layer set merging, the YOLOv5s-RMV3S method is constructed. Its computational volume drops to 6.3 GB and its model size drops to 6.8 MB (model size drops by 62% and accuracy rate drops by 1.1 percentage points). It can be seen that after improving the backbone extraction network structure and loss function, a high recognition accuracy is ensured with a significant reduction in computation, number of parameters, and model size, which not only meets the lightweight requirements of air control and airspace management, but it also provides sufficient preparation for the subsequent Auto-SD and Ad-EDB, and some of the detection results are shown in Figure 8.

As can be seen from Figure 8, the algorithm improved by this paper achieves better detection results for helicopters, while still achieving a high confidence level when the helicopter is distant or partially missing overall. Using test videos for validation, the object detection module in this paper makes each frame of the image complete with helicopter cropping, so the object detection module ensures recognition accuracy while significantly reducing model size and computational effort, saving subsequent model loading time and generating the original data image data set.

The experimental environment in this paper is: NVIDIA Tesla V100 made in Santa Clara, CA, USA, 8 GB RAM, Linux 16.04 OS, PyTorch version 1.10.2 (Linux Foundation, San Francisco, CA, USA), torchvision version 0.11.3, and CUDA version 11.1 (NVIDIA).

### 4.2. Comparison of Experimental Results

#### 4.2.1. Experimental Results on Self-Learning Mechanisms

In terms of the construction of Auto-SD, this paper hopes to select a suitable threshold β to complete the selection of core stationary position labels, so as to achieve the state classification of the data. Therefore, this paper has conducted a comparison experiment in a multi-segment, multi-camera, and multi-scene helicopter entry and departure video scene to observe the performance comparison of different thresholds on positive and negative sample selection, and the results are shown in Table 2.

The following conclusions can be drawn from Table 2, where the threshold β starts at 0.92, and the selection accuracy increases as the threshold value increases. The accuracy of positive and negative sample selection peaked when the threshold β was at 0.95, obtaining an accuracy rate of 91.32%, and then as the threshold β decreased, the selection accuracy became smaller. The threshold value β had less influence on the selection accuracy when the fluctuation was small. Therefore, this paper sets the threshold β to 0.95 and determines the number of frames corresponding to the core stationary position label to achieve the self-generation of image data state labels.

**Figure 9:**
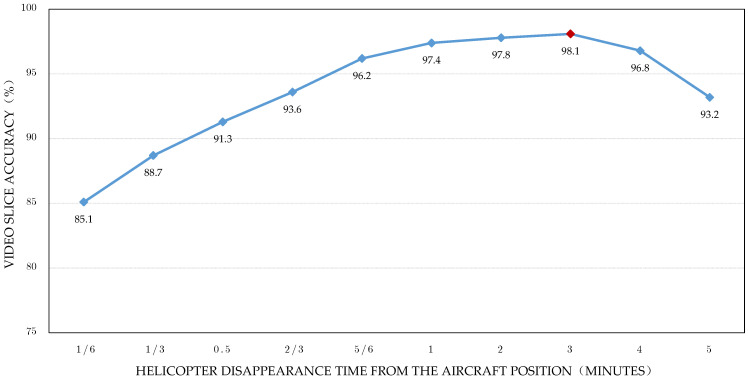
Comparison of the effect of vanishing time length on cutaway video.

The live heliport surveillance position recordings are lengthy and contain multiple helicopter entries and departures. In the construction of Auto-SD, this paper wishes to select a suitable time window for slicing the live video such that only one segment of the helicopter in the off-position state can be analyzed at a time. By counting the results of the object detection, the real-time movement of the helicopter in the helicopter position monitoring area can be obtained, and then the length of time for which the helicopter disappears in the position area can be obtained based on the statistics of the video frames. Therefore, the length of time for which the helicopter disappears from the aircraft area is chosen as a measure in this paper. However, due to the hovering nature of the helicopter, the mere fact that the helicopter is detected in the video and remains stationary does not fully equate to the completion of helicopter entry and departure. According to the helicopter flight manual, helicopter hovering time should not exceed 3 min, which provides the theoretical basis for this experiment. As shown in Figure 9, experiments were conducted on multiple helicopter entry and departure videos containing multiple segments, each of which was up to 24 h long, and each of which contained at least 12 segments of the helicopter entry and departure process. Setting the vanishing time length to 3 min was compared with other vanishing time lengths, and the experiments showed that there was no major difference in the effect of setting the vanishing time length to 1–3 min, but the 3 min cutoff was the best.

**Figure 10:**
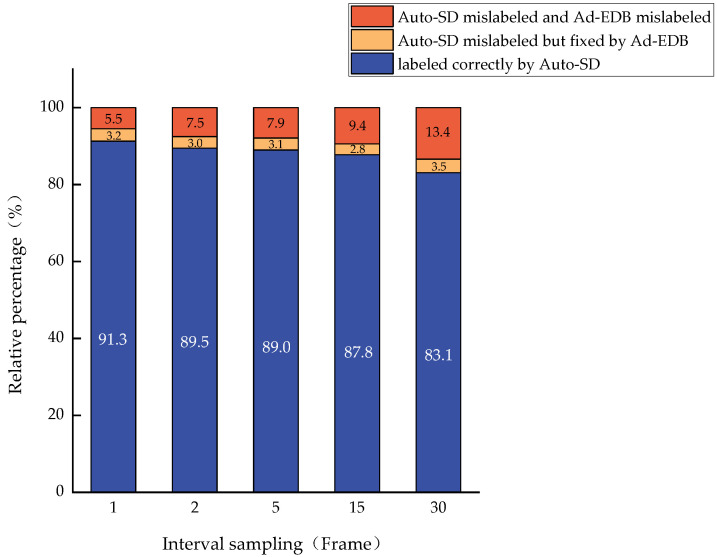
Comparison of the generation results of Auto-SD and Ad-EDB.

**Figure 11:**
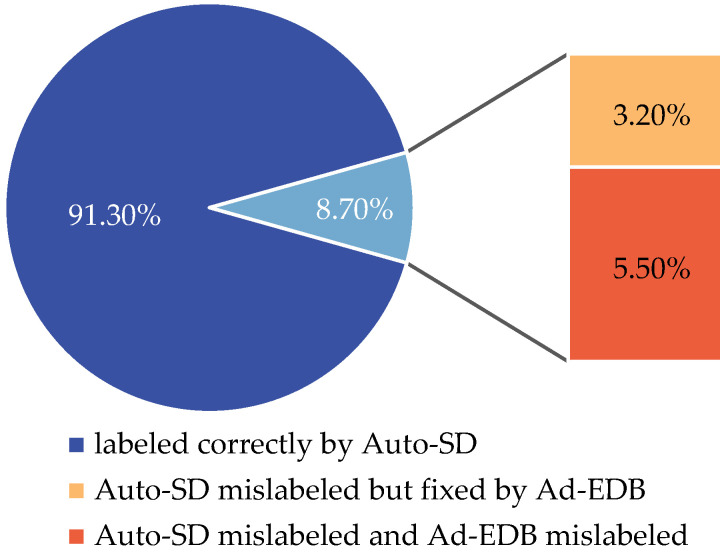
Specific generation occupancy and repair occupancy for a frame interval of 1.

We can automatically generate the corresponding status labels through the proposed Auto-SD and Ad-EDB. To verify the validity and accuracy of our methods, 10 intercepted complete entry and departure videos are selected as the data set, and accurate motion state labels were manually created on the video frames. These manually created state labels are compared with those generated by Auto-SD and Ad-EDB. In this case, the inter-frame information is sampled at different length intervals and repeated 10 times to find the average of the accuracy rates. The specific generation percentage and repair percentage are shown in Figure 10 and Figure 11.

**Figure 12:**
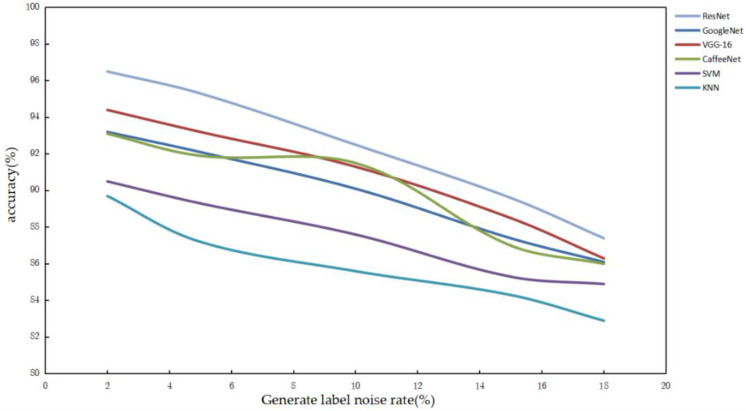
Comparison of the noise immunity performance of various image classification algorithms.

In order to ensure the matching effect of the constructed Ad-EDB in selecting the image classifier according to the noise rate of the generated labels, this paper processed the original correctly labeled data set. No more than 20% of the labels were randomly selected for image classification after alteration in the experiments, and the experimental results were averaged and analyzed. The correlation between the generated label noise rate and the accuracy of each image classification algorithm was observed experimentally, and the experimental results are shown in Figure 12.

As can be seen from Figure 12, ResNet has good data noise immunity and can guarantee about 95% image classification accuracy under the influence of a 5% noise rate. CaffeeNet shows small fluctuations in accuracy when generating label noise rates of 6–10%, but its results are within the boundary error tolerance, and the data are still weaker than ResNet in terms of noise immunity; the experimental error is negligible. Other convolutional image classification algorithms such as GoogleNet and VGG-16 are weaker than ResNet in terms of noise immunity in image classification. Machine learning methods such as SVM and KNN have poor noise immunity, and are unable to meet the demand for image classification accuracy in the problem of helicopter entry and departure recognition.

#### 4.2.2. Experimental Results of the Overall Algorithm

As listed in Table 3, comparative experiments were conducted on live videos of helicopter flight conditions in multiple scenes and multiple aircraft positions in order to verify the conditioning capability of Ad-EDB and the accuracy of the overall algorithm in this paper. At the same time, the entry and departure false detection rates were introduced because the movement of the helicopter during detection is difficult to determine. The entry false detection rate is the probability of mistakenly detecting an entry as an exit during inspection, and the departure false detection rate is the probability of mistakenly detecting an exit as an entry during inspection. In this paper, the actual entry time is defined as $t_{1}$, the actual departure time as $t_{2}$, the detection entry time as $t_{e}$ and the detection departure time as $t_{d}$. If $\left|t_{e}-t_{1}\right|\geq 2s$, it is assumed that the detection will be entry false. If $\left|t_{d}-t_{2}\right|\geq 2s$, it is assumed that the detection will be departure false. SVM-based and Random Forest-based methods are subject to large fluctuations in noise, and are less accurate and slower. In the generated bounding box method, the helicopter entry and departure times are judged directly based on the IOU situation between each detected bounding box, but only 91.32% accuracy is achieved because the bounding boxes jitter during detection, and the image context information is ignored, with the entry and departure false detection rates being 5.15% and 3.53%, respectively. Due to the agility and real-time nature of the sensor, the laser method can achieve an accuracy of 95.35% in detection, but the problem presented in this paper cannot be solved, as the sensor is not built for general aviation airports and the equipment is too expensive to complete self-learning. The propeller rotation method only relies on the rotation of the propeller to determine the entry and departure of the helicopter, ignoring the in situ information of the helicopter and its own movement, achieving an accuracy rate of 83.21%, of which the entry and departure false detection rates are 9.43% and 7.36%, respectively. The algorithm in this paper achieves an accuracy of 97.83% for the entry and departure position identification, which is a 6.51 percentage point improvement compared to the generated bounding box method. The tags are then identified after Ad-EDB adjustment, with a 2.12 percentage point improvement in accuracy compared to when they are not adjusted. Meanwhile, this paper can complete self-learning through the constructed Auto-SD with Ad-EDB, which significantly alleviates the strong reliance on the manual annotation and manual selection of training samples for the helicopter entry and departure problem.

## 5. Conclusions

In order to obtain accurate helicopter entry and departure times, this paper constructs a helicopter entry and departure recognition method with a self-learning mechanism as the core, supplemented by a lightweight object detection module and an image classification module. Using the original image information obtained by the object detection module as input to the self-learning mechanism, Auto-SD and Ad-EDB are designed and built to automatically generate image data motion state labels and complete the adjustment and evaluation, and then select the best image classification module to complete the helicopter entry and departure state recognition. It uses the pre-order classification results to continuously optimize the annotation of the video to be detected in the post-order, forming a self-learning closed loop in the algorithm. In this paper, we hope to build more general and generalizable self-learning mechanisms in order to carry out subsequent research on the generalizability and effectiveness of self-learning mechanisms in more diverse business scenarios.

## Figures and Tables

**Table 1:** Comparative experimental results of the improved process of the object detection module.

Method	P/%	R/%	mAP/%	Parameters/M	Computation/G	Size/MB
YOLOv5s	97.4	97.1	97.4	7.1	16.4	13.7
YOLOv5s-RMV3S	96.3	96.1	96.3	3.5	6.3	6.8

**Table 2:** Comparison of the performance of different thresholds for positive and negative sample selection.

Threshold β	Selection Accuracy/%
0.92	90.31
0.93	90.53
0.94	91.13
0.95	91.32
0.96	90.55
0.97	90.37

**Table 3:** Performance comparison of the overall algorithm.

Method	Entry False Detection Rate/%	Departure False Detection Rate/%	Recognition Accuracy/%	Availability of Self-Study
SVM-based method	7.12	4.15	88.73	No
Random Forest-based method	6.03	4.01	89.96	No
YOLOv5s (generating bounding boxes)	5.15	3.53	91.32	No
Laser method (application sensors)	3.15	1.50	95.35	No
Optical flow method (determining propeller motion)	9.43	7.36	83.21	No
Lightweight Object Detection + Auto-SD + ResNet18	2.94	1.35	95.71	No
Lightweight Object Detection + Auto-SD + ResNet18 + Ad-EDB	1.15	1.02	97.83	Yes

## Data Availability

Not applicable.
